# Taxonomic and functional assembly cues enrich the endophytic tobacco microbiota across epiphytic compartments

**DOI:** 10.1128/msphere.00607-23

**Published:** 2023-12-12

**Authors:** Luhua Yang, Yuan Guo, Hui Yang, Shun Li, Yunzeng Zhang, Likai Hao

**Affiliations:** 1 Key Laboratory of Urban Environment and Health, Ningbo Urban Environment Observation and Research Station, Institute of Urban Environment, Chinese Academy of Sciences, Xiamen, China; 2 Zhejiang Key Laboratory of Urban Environmental Processes and Pollution Control, CAS Haixi Industrial Technology Innovation Center in Beilun, Ningbo, China; 3 State Key Laboratory of Environmental Geochemistry, Institute of Geochemistry, Chinese Academy of Sciences, Guiyang, China; 4 University of Chinese Academy of Sciences, Beijing, China; 5 Guizhou Academy of Tobacco Science, Guiyang, China; 6 College of Bioscience and Biotechnology, Yangzhou University, Yangzhou, China; 7 CAS Center for Excellence in Quaternary Science and Global Change, Xi’an, China; E.O. Lawrence Berkeley National Laboratory, Berkeley, California, USA

**Keywords:** plant microbiome, endophytes, endosphere, community assembly, secondary metabolites

## Abstract

The plant microbiome plays a critical role in plant growth, development, and health, with endophytes being recognized as essential members due to their close interactions with host plants. However, knowledge gaps remain in understanding the mechanisms driving the colonization and establishment of endophytic communities. To address this issue, we investigated the microbiota of tobacco roots and leaves, including both epiphytic and endophytic microorganisms. We found that Actinobacteria and Alphaproteobacteria were significantly enriched in the root endosphere. Additionally, we identified higher abundances of functional traits involved in antibiotic synthesis, plant cell wall degradation, iron metabolism, secretion systems, and nicotine degradation enzymes in the endosphere. We further studied metagenome-assembled genomes from the rhizosphere and root endosphere, revealing a greater diversity of secondary metabolites in bacteria within the root endosphere. Together, this study provides insights into the taxonomic and functional assembly cues that may contribute to shaping the endophytic plant microbiota.

**IMPORTANCE:**

The presence of diverse microorganisms within plant tissues under natural conditions is a well-established fact. However, due to the plant immune system’s barrier and the unique microhabitat of the plant interior, it remains unclear what specific characteristics bacteria require to successfully colonize and thrive in the plant endosphere. Recognizing the significance of unraveling these functional features, our study focused on investigating the enriched traits in the endophytic microbiota compared to the epiphytes. Through our research, we have successfully identified the taxonomic and functional assembly cues that drive the enrichment of the endophytic microbiota across the epiphytic compartments. These findings shed new light on the intricate mechanisms of endophyte colonization, thereby deepening our understanding of plant-microbe interactions and paving the way for further advancements in microbiome manipulation.

## INTRODUCTION

Plants host a wide array of microorganisms, which can be categorized as epiphytes, residing on the plant’s surface, and endophytes, inhabiting the interior of plants (1). The epiphytic microbiota is believed to be less influenced by host innate immune signaling (2), while the endophytic microbiota is supposed to have more intimate interactions with plant hosts as they could evade the plant immune system (3, 4). A lower diversity and a higher degree of specialization have been found toward the root interior (5, 6). While a plethora of literature has characterized the plant microbiome of many species (7–10), the functional distinctions between epiphytic and endophytic compartments have rarely been addressed. Although some efforts have been made to explore the genomic features of root microbiota (11, 12), these studies often either solely focused on the rhizosphere or failed to differentiate the endosphere from the rhizosphere. A comprehensive profiling and comparison of epiphytic and endophytic compartments is still missing, limiting our understanding of the features necessary for the colonization and establishment of endophytes.

Recent studies have revealed that endophytes are reservoirs of bioactive secondary metabolites (13), which are not essential for regular cell growth but confer a competitive advantage to the producer (14). Secondary metabolites play important roles in facilitating intercellular communication, inhibiting competitors, aiding in nutrient acquisition, and shaping interactions with the surrounding environment (15). While most research has concentrated on the potential applications of secondary metabolites derived from endophytes, relatively few have delved into the significance of secondary metabolites in endophyte colonization. Thus, the correlation between secondary metabolites and the assembly of the endophytic community remains elusive.

In this study, we systematically investigated the microbiome associated with tobacco (*Nicotiana tabacum*). By investigating the endophytic and epiphytic compartments in both root and leaf samples, we attempted to reveal the taxonomic and functional features enriched in the plant endosphere in comparison with the epiphytic compartments, with a focus on secondary metabolites using genome mining. Our results aimed to shed light on the assembly rules and colonization mechanisms of plant endosphere microbiota.

## MATERIALS AND METHODS

### Sample collection and DNA extraction

Three phylogenetically related varieties of tobacco (*Nicotiana tabacum*) were selected for investigation, namely, K326, Y87, and Y28. Variety K326 serves as the parent of variety Y87, and variety Y87, in turn, is the parent of variety Y28 (Fig. S1, Supporting Information). We chose these three varieties because closely related host species typically display a higher degree of similarity in their associated microbiota, as demonstrated in previous research (10, 16, 17). Since our main objective is to make a comparison between the endophytic and epiphytic compartments, we deliberately opted for phylogenetically related varieties rather than randomly chosen cultivars. This approach enables us to reduce the potential impact of significant differences among the varieties.

The three varieties were cultivated in adjacent but separate fields within the tobacco-farming region of Tianma, Anshun, and Guizhou, China (26°24′19.548″N, 106°15′24.588″E), using the same agricultural practices. The tobacco plants underwent standard fertilization and pesticide applications. However, no fertilizers, pesticides, or herbicides were applied for 2 weeks prior to the sampling. No growth differences were observed among the three varieties; in other words, all three were at the same developmental stage.

Destructive sampling was conducted during the topping stage, around 10 days after the removal of terminal buds. Specifically, the bulk soil, roots, and top leaves were collected on 26 July 2020. Triplicates were sampled for each variety and were transferred to the laboratory on ice immediately. The soil cores (at 0–20 cm depth) from each field were collected and pooled as one bulk soil replication, with each containing around 80 g. The plants were further divided into root rhizosphere, root endosphere, leaf epiphytes, and leaf endophytes, as described below. The loosely attached soil was removed by shaking. The roots were then put into a 50-mL flask with phosphate buffered saline (PBS) buffer and washed on a shaking platform for 20 min at 180 rpm. The washing buffer was subjected to centrifugation (1,500 × *g*, 20 min), and the resultant pellet was defined as a rhizosphere compartment. The roots were then transferred to a new 50-mL Falcon tube. After a second washing step (20 min, 180 rpm) with PBS buffer and surface sterilization with 75% ethanol (5 min, 180 rpm), roots were flash frozen using liquid nitrogen and defined as the root compartment (root endosphere). The leaves were treated in the same way as the roots.

All compartments were flash frozen by liquid nitrogen and stored at −80°C. The DNA was extracted using the Mag-MK Soil Genome DNA Extraction Kit (Sangon, China). In total, we got 45 samples, including 3 varieties and 5 compartments.

### Amplicon and metagenome sequencing

The primer pairs 799F and 1193R (18) were used for the bacterial 16S rRNA gene amplification to avoid the co-amplification of chloroplast DNA. The details of PCR amplification and library preparation were described in the supplementary methods section of the Supporting Information. The amplicon libraries were sequenced on the Illumina Nova6000 platform (Illumina, USA).

For the metagenome sequencing, the genomic concentrations were quantified using the Qubit dsDNA HS Assay Kit (Thermo Fisher Scientific, USA). Libraries with an insert length of about 500 bp were prepared with a total of about 500 ng of DNA. The DNA of each sample was mechanically sheared to around 500 bp fragments using Covaris S220 (Covaris, USA). The sheared DNA was then processed with the NEBNext Ultra DNA Library Prep Kit for Illumina (New England Biolabs, USA) for end repair and adaptor ligation. The fragmented DNA was recovered with 1× Hieff NGS DNA Selection Beads (Yeasen Biotechnology, China). The purified PCR products were assayed using a Qubit 4 fluorometer (Thermo Fisher Scientific, USA) and sequenced on the Illumina Hiseq platform (Illumina).

### Bioinformatics

#### Quality control, denoise, and taxonomy annotation of 16S rRNA amplicon sequencing

The amplicon sequencing analysis was performed with the software Usearch (v11.0.667). After the removal of primers and Phix contamination, the reads were merged and filtered. The clean reads were denoised using the UNOISE3 algorithm (19). The denoised sequences, which are the correct biological sequences in the reads, are called "zOTUs" (zero-radius OTUs). The taxonomy assignment of the 16S rRNA gene was performed using the RDP training set (v18) (20) with the SINTAX taxonomy prediction algorithm (21). Reads that were assigned to chloroplast and mitochondria were filtered out.

#### Quality control and reads-based analysis of metagenome sequencing

For metagenome sequencing, the quality control and removal of plant-derived reads were performed by KneadData (v0.10.0) (http://huttenhower.sph.harvard.edu/kneaddata). On average, the percentage of clean reads in each plant compartment was 85.97% for the bulk soil (ranging from 86.96% to 89.70%), 73.65% for the rhizosphere (ranging from 56.29% to 81.31%), 11.93% for the root endosphere (ranging from 3.03% to 28.33%), 1.65% for the leaf episphere (ranging from 0.82% to 3.10%), and 0.12% for the leaf endosphere (ranging from 0.02% to 0.28%). The taxonomy was assigned using MetaPhlAn (v3.0.13) (22). The functional profiling was carried out with HUMAnN (v3.0) (22).

#### Metagenome assembly and functional annotation

The metagenomic reads were assembled by MEGAHIT (v1.2.9) (23). The assembled contigs were taxonomically classified using MMseqs2 (v13.45) (24). Reads assigned to eukaryotes, viruses, and archaea were discarded. Gene annotation, clustering, and quantification were performed with Prodigal (v1.14.6) (25), MMseqs2 (v13.45) (24), and CoverM (v0.3.2) (https://github.com/wwood/CoverM), respectively. The functional annotation was conducted with eggNOG (v5.0) against eggNOG-Mapper (v2.0.1) (26). Particularly, we focused on the annotation of Kyoto Encyclopedia of Genes and Genomes (KEGG) modules, where each gene or protein was assigned a KEGG Orthology (KO).

#### Metagenome binning, genome annotation, and secondary metabolite prediction

The genome reconstruction was conducted with MetaWRAP (v1.2) (27). Metagenome-assembled genomes with completeness ≥70% and contamination ≤10% were considered to have high qualities and were retained for further refining using MetaWRAP (v1.2). The refined bins were dereplicated using dRep (v3.4.0) (28). The taxonomy of the bins was annotated by GTDB-Tk (v2.1.1) (29). The prediction of biosynthetic gene clusters in metagenome-assembled genomes was performed using antiSMASH (v6.1.1) (30) with the default setting.

### Statistical analysis and visualization

#### Alpha and beta diversity analysis

The original data of the three varieties were used for the independent diversity analysis, that is, without combination. The alpha diversity was calculated with the R package “hilldiv” (31) using Hill numbers as the diversity index. The beta diversity was visualized in the principal coordinate analysis (PCoA) plot using the Bray-Curtis dissimilarity generated by the Usearch pipeline. The permutational multivariate ANOVA was tested using the method *adonis* in R package “vegan” (32) with 999 times permutation.

#### Construction and visualization of co-occurrence networks

The microbial networks were constructed using the Molecular Ecological Network Analyses Pipeline (33). From this step on, the data from the three varieties were pooled for further analysis. Four molecular ecological networks were constructed, each representing one compartment. Only OTUs present in no less than half of the samples were included in the correlation calculation. Pearson correlations were performed with log-transformed OTU abundances. The cut-off threshold was determined by the Random Matrix Theory based method, and the default values were adopted for network construction. The indirect relationships were removed using the iDIRECT program (34). The degree of community complexity was quantified using the metric cohesion, and the calculation was performed using the ‘taxa shuffle’ null model with the R code provided by the author (35). The networks colored by taxonomy were visualized using the software Gephi (36) with the Fruchterman-Reingold layout. The networks colored by modules were visualized with the R package “igraph” (37).

#### Analysis of community assembly processes

The modified stochasticity ratio (MST), a metric to estimate ecological stochasticity according to a null-model-based statistical framework, was calculated using the R package “iCAMP” (38). The MST index value of 50% was chosen as the dividing point between deterministic-dominated (<50%) and stochastic-dominated (>50%) community assembly processes (39).

#### Identification of possible OTU sources in each compartment

We conducted source tracking analysis to estimate the contribution of potential sources to the plant microbiome in each compartment. The microbiome in the target compartment was considered a “sink,” while the microbiome in the soil, air, or adjunct plant compartment was considered a possible “source” for the sink. The fraction of various source microbial communities for a given sink in each plant compartment was estimated using the software FEAST (Fast Expectation-maximization for microbial Source Tracking) (40) based on the OTU matrix.

#### Identification of differentially abundant OTUs between endophytic and epiphytic compartments

The data from the three varieties were pooled to identify the enriched taxa and functions. Based on the results of amplicon sequencing, we compared the taxonomy differences between the endophytic and epiphytic compartments in root and leaf samples using the R package “ALDEx2” (ANOVA-Like Differential Expression tool for high-throughput sequencing data) (41). The comparison was performed at the level of class, order, family, and genus. We used *Aldex2* because it was proven to produce the most consistent results across studies and agrees best with the intersection of results from different approaches in amplicon data sets (42). The outputs were considered significant when the parameters we.eBH <0.05, wi.eBH <0.05, and overlap <0.05 were met. The parameter we.eBH denotes the Expected Benjamini-Hochberg corrected *P* value of Welch’s *t*-test. The parameter wi.eBH denotes the Expected Benjamini-Hochberg corrected *P* value of the Wilcoxon test. The term overlap means the proportion of effect size that overlaps 0 (i.e., no effect).

As to the comparison between endophytic and epiphytic compartments based on metagenome sequencing, we used Linear discriminant analysis Effect Size (LEfSe) (43) on the Galaxy platform (http://huttenhower.sph.harvard.edu/galaxy). LEfSe is a widely recognized algorithm for high-dimensional biomarker discovery and explanation, primarily tailored for metagenome sequencing. It first conducts the Kruskal-Wallis test to choose features differentially distributed among groups, then applies the pairwise Wilcoxon test to the retained features, and finally provides Linear Discriminant Analysis (LDA) bootstrapping support. Features were considered significant if they had a *P* value <0.05 in both the Kruskal-Wallis test and the Wilcoxon test, as well as an LDA score >2. The analysis was done at phylum, class, order, family, and genus levels, and the final output was illustrated in a phylogenetic tree.

#### Identification of differentially abundant KEGG Orthologs

The data from the three varieties were pooled to identify the enriched functions. In this study, we used KO to denote functional orthologs. The enriched functional KOs in the endophytic compartment in comparison with the epiphytic compartment were determined using R package “DESeq2” (44). DESeq2 first applies normalization to account for differences in library size and composition between samples, then models the distribution of read counts using a negative binomial distribution, and estimates the dispersion of counts for each KO. It then conducts a hypothesis test (the Wald test) to identify KOs that are differentially abundant between conditions. The false discovery rate was applied for correction. The outputs of these tests include log2-fold changes and *P* values for each KO. Results with an adjusted *P* value <0.05 and an absolute value of log2-fold change >1.5 were considered significant.

#### Visualization of metagenome-assembled genomes

The phylogenetic tree of the refined metagenome-assembled genomes was constructed and visualized using iTOL (v6) (45, 46).

## RESULTS

### The tobacco microbiome is mainly shaped by compartment niches

**FIG 1:**
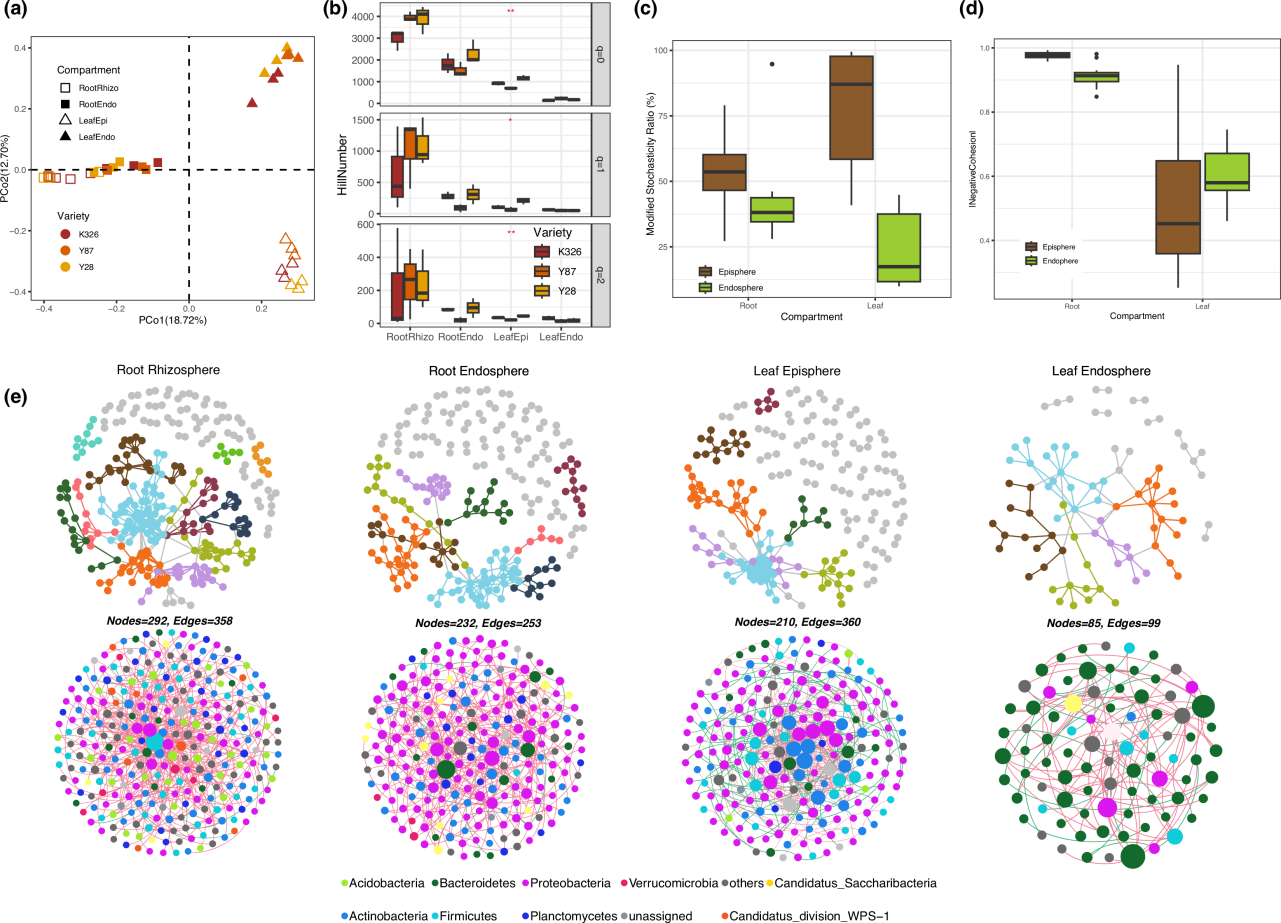
(a) The PCoA plot suggests that the tobacco microbiome is primarily influenced by compartment niches. Each compartment is distinguished by distinct shapes, while variety is represented by various colors. Squares denote the root compartment, triangles represent the leaf compartment, solid shapes signify the epiphytic compartment, and hollow shapes refer to the endophytic compartment. (b) The alpha diversity of tobacco microbiome in each compartment is presented with Hill numbers in different order of *q*. The varieties are represented by different colors. The alpha diversity decreases from the root rhizosphere to the root endosphere and leaf compartment. Differences in alpha diversity among the varieties are indicated with asterisks (*P < 0.05, **P < 0.01). (c) The Modified Stochasticity Ratio (MST) of the tobacco microbiome in the root and leaf compartments is calculated. Epiphytic samples are represented by the brown color, while endophytic samples are depicted in green. MST serves as an index, designed with a 50% threshold to distinguish between more deterministic (<50%) and more stochastic (>50%) processes in the community assembly. (d) The complexity of microbial co-occurrence networks was assessed using the index of cohesion. The figure illustrates the absolute value of the negative cohesion within microbial co-occurrence networks of both root and leaf compartments. Brown is used to denote epiphytic samples, while green represents endophytic samples. (e) A visualization of the constructed microbial co-occurrence networks within each compartment is presented. In the top row, nodes are colored based on modules, with large modules containing ≥5 nodes displayed in distinct colors, while modules with less than 5 nodes are depicted in gray. In the bottom row, nodes are colored by phylum, as explained in the legend. Note: The number of nodes and edges in the networks within the same compartment is the same, as identical data were used as input in both cases. The visual differences between the networks in the top and bottom rows mainly result from the choice of visualization software. The networks colored by modules were visualized with the R package igraph, whereas the networks colored by taxonomy were visualized using the software Gephi.

Our results indicated that the tobacco microbiome was mainly shaped by compartment niches (*R*^2^ = 40.48%, *P* < 0.001). The influence of varieties, although statistically significant (*P* < 0.05), was relatively modest, explaining 5.28% of the variance (*R*^2^ = 5.28%), as revealed by permutational multivariate analysis of variance (with *R*^2^ indicating the factor’s contribution to the variance). Accordingly, the plant microbiome was clearly separated by compartments in the PCoA plots, whereas the clustering of varieties was less prominent (Fig. 1a).

We further assessed the alpha diversity of tobacco bacterial communities. The Hill number was used as the unified diversity index, as it could represent richness, the Shannon index, and the Simpson index with the scaling parameter *q*. Results suggested that the compartment was the major factor influencing the alpha diversity, while the influence of the variety was only observed in the epiphytic leaf compartment (Fig. 1b). The diversity of the tobacco microbiome declined from the rhizosphere to the root endosphere and from roots to leaves. The leaf epiphytes showed higher diversity than leaf endophytes only when *q* number equals 0 (insensitive to OTU frequencies and equivalent to richness) (Fig. 1b).

The MST was calculated to estimate the community assembly process of each compartment. Notably, the MST ratios of both root endophytes and leaf endophytes were below 50% (Fig. 1c), indicating that the assembly of the endosphere compartments was dominated by a deterministic process. In contrast, the MST ratio of epiphytic compartments was much higher than that of the endophytic compartment, suggesting that stochastic processes played more important roles in the community assembly of the epiphytic compartment.

We then investigated the patterns of co-occurrence networks. The degree of community complexity was quantified using the metric cohesion (Fig. 1d). Results indicated that the absolute value of cohesion was highest in the rhizosphere, followed by the root endosphere and phyllosphere, as well as the alpha diversity. However, no statistical differences were observed between leaf epiphytes and leaf endophytes (Fig. 1d). Clearly, the taxonomic composition of the networks differed among plant compartments. Notably, a large proportion of nodes assigned to Bacteroidetes were observed in the leaf endosphere (Fig. 1e).

### Taxa are selectively enriched in the endosphere compartment

We then conducted source-tracking analysis to estimate the contribution of potential sources to the plant microbiome in each compartment. The microbial communities were mainly derived from the neighboring compartments, and the microbiome was gradually filtered from the epiphytic to the endophytic compartment (Fig. S2, Supporting Information). The bulk soil only accounted for a small proportion of leaf epiphytes, indicating that the major source of the phyllosphere might be contributed by other environmental sources, such as the air.

**FIG 2:**
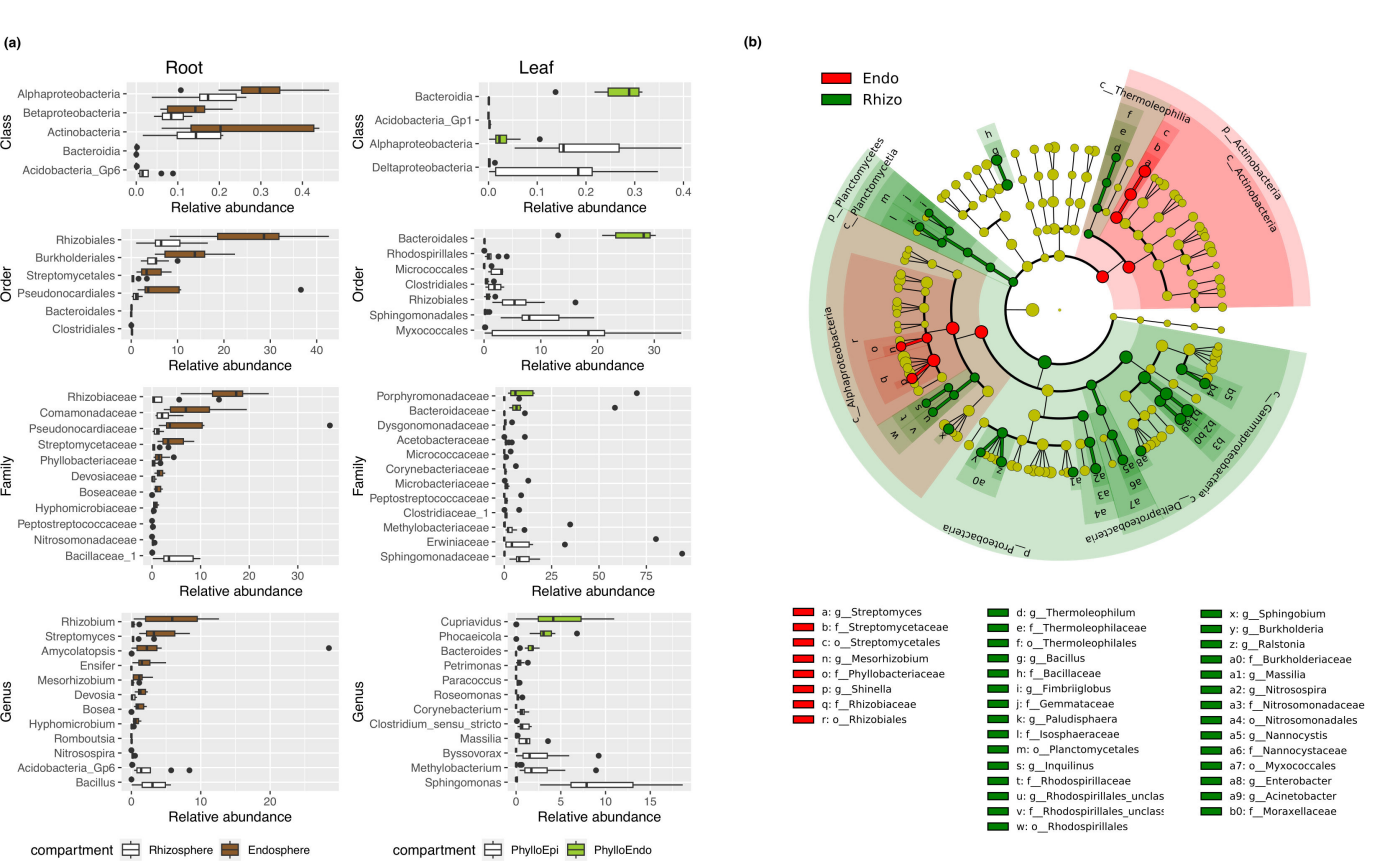
(a) In the amplicon sequencing, taxa with statistically significant differences in abundance between the endophytic and epiphytic compartments were identified at the class, order, family, and genus levels. The epiphytic compartments of both the root and the leaf were depicted in white. The root endosphere was represented in brown, while the leaf endosphere was distinguished by the color green. The boxplot shows the distribution of the relative abundance of the taxa. (b) In the metagenome sequencing, taxa with different abundances between the root endosphere and the rhizosphere were depicted in the phylogenetic tree. The phylum and class enriched in the endosphere were highlighted with a red shadow, while the phylum and class enriched in the rhizosphere were highlighted with a green shadow. Red nodes represent taxa enriched in the root endosphere at the order/family/genus level. Green nodes represent taxa enriched in the rhizosphere at order/family/genus level. The yellow nodes denote taxa with no statistical differences.

To elucidate which taxa were enriched or depleted in the endosphere, we conducted differential abundance analysis between the endophytic and epiphytic compartments. The results based on amplicon sequencing indicated that Actinobacteria (including *Streptomyces* and *Amycolatopsis*) and Alphaproteobacteria (including *Rhizobium*, *Ensifer*, *Mesorhizobium*, *Devosia*, *Bosea*, and *Hyphomicrobium*) were enriched in the root endosphere (Fig. 2a). In contrast, *Bacillus* and *Romboutsia* in Firmicutes, *Nitrosospira* in Nitrospira, and *Gp6* in Acidobacteria were found to be more abundant in the rhizosphere (Fig. 2a). In the phyllosphere, however, different patterns were observed. Alphaproteobacteria, including *Sphingomonas* and *Methylobacterium*, was strongly depleted from the leaf endosphere. On the contrary, Bacteroidetes (including *Phocaeicola*, *Bacteroides*, and *Petrimonas*) was enriched in the leaf endosphere (Fig. 2a).

Results based on the metagenome sequencing indicated that Alphaproteobacteria and Actinobacteria were enriched in the root endosphere (Fig. 2b), which was consistent with the results of amplicon sequencing. But no statistical differences were detected in the phyllosphere.

### Functions are selectively enriched in the endosphere compartment

**FIG 3:**
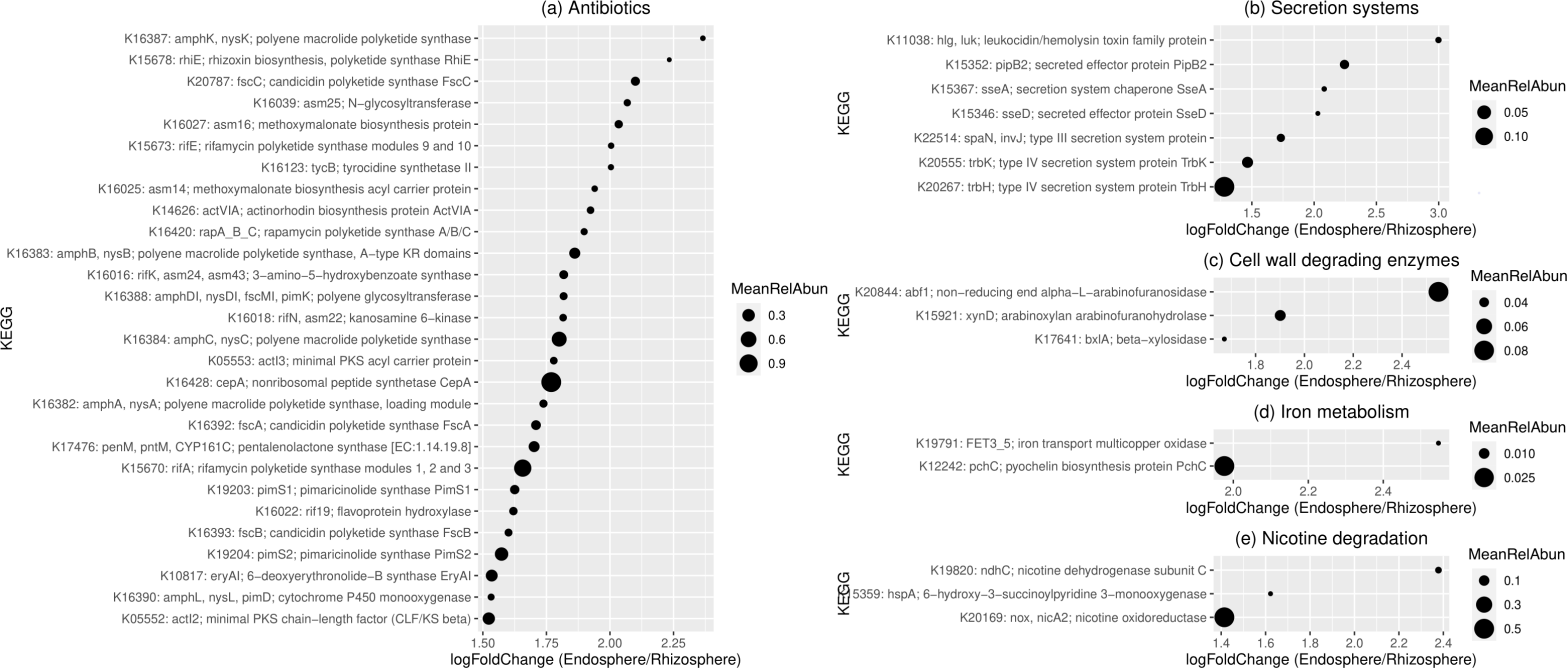
The KOs are enriched in the root endosphere in comparison with the rhizosphere based on the results of metagenome sequencing. The x axis indicates the log2-fold change of the corresponding KO. The size of the dot was in accordance with the mean abundance of the KO. The enriched KOs are involved in (a) antibiotic synthesis, (b) secretion systems, (c) cell-wall-degrading enzymes, (d) iron metabolism, and (e) nicotine degradation.

No statistical differences were detected between the endo- and epiphytic leaf compartments. In contrast, a total of 232 KOs were found to be enriched in the root endosphere in comparison with the rhizosphere. Notably, more than one-eighth of the enriched KOs were involved in antibiotic synthesis, including tetracycline, rhizoxin, candicidin, ansamycins, rifamycin, tyrocidine, actinorhodin, rapamycin, erythromycin, pentalenolactone, pimaricin (natamycin), and vancomycin (Fig. 3a). Most of the antibiotics were synthesized using polyketide synthases. Non-ribosomal peptide synthetase (NRPS) CepA (K16428) was also detected and was the most abundant among the enriched KOs engaged in antibiotic synthesis.

Seven enriched KOs were involved in secretion systems, where four belonged to type III secretion systems and two belonged to type IV secretion systems (Fig. 3b). Intriguingly, the most abundant KO, type IV secretion system protein TrbH (K20267), was assigned to Alphaproteobacteria.

Three KOs related to plant cell wall degrading enzymes were enriched in the root endosphere, which were non-reducing end alpha-L-arabinofuranosidase (K20844), arabinoxylan arabinofuranohydrolase (K15921), and beta-xylosidase (K17641) (Fig. 3c). The alpha-L-arabinofuranosidase is involved in the degradation of xyloglucan, a hemicellulose that occurs in the primary cell wall of all vascular plants. The arabinofuranohydrolases have been implicated in the degradation of arabinoxylan, a hemicellulose found in both the primary and secondary cell walls of plants. Beta-xylosidase is a pivotal enzyme for the complete degradation of xylan, the second main constituent of plant cell walls. Among the three enzymes, the non-reducing end alpha-L-arabinofuranosidase was the most abundant and showed the largest fold change between the endo- and epiphytic root compartments.

KOs in the pathway of iron metabolism were found to be enriched in the root endosphere (Fig. 3d). One ortholog was iron transport multicopper oxidase (K19791), which is required to oxidize ferrous iron to ferric iron for further iron transport. The other ortholog was pyochelin biosynthesis protein (K12242), which is a siderophore chelating iron.

Intriguingly, three KOs enriched in the root endosphere were associated with the degradation of nicotine (Fig. 3e), the main alkaloid produced in tobacco, including nicotine dehydrogenase (K19820), 6-hydroxy-3-succinoylpyridine 3-monooxygenase (K15359), and nicotine oxidoreductase (K20169).

### Secondary metabolites predicted in the metagenome-assembled bins

**FIG 4:**
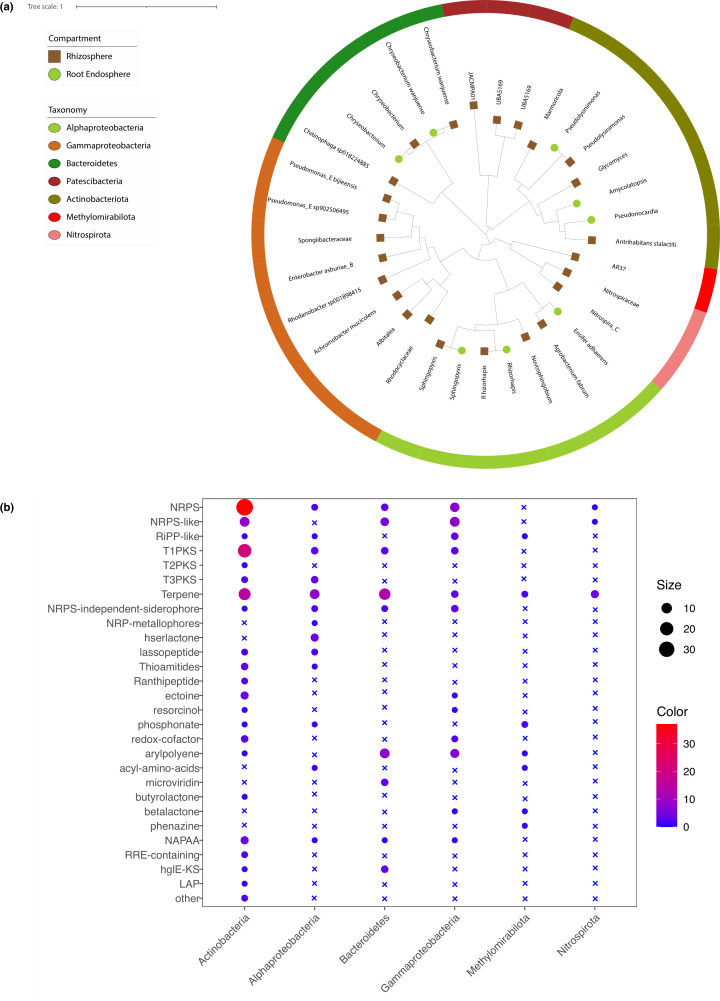
The diversity of secondary metabolites in the metagenome-assembled genomes retrieved from the root endosphere exceeded that found in the rhizosphere. (a) The phylogenetic tree displays metagenome-assembled genomes obtained from both the rhizosphere and the root endosphere. Each node represents a metagenome-assembled genome, with node color indicating its source: brown for the rhizosphere and green for the endosphere. Additionally, the color of the outer ring reflects the taxonomy of each genome at the class level. (b) The predicted secondary metabolites within metagenome-assembled genomes. Genomes belonging to the same class were combined for evaluation. The cross symbol is used to signify the absence of secondary metabolites, while the size and color of the circles correspond to the abundance of these metabolites.

A total of 61 and 22 high-quality metagenome-assembled bins were obtained from the rhizosphere and root compartment, respectively. Further dereplication resulted in 25 unique bins in the rhizosphere and 8 unique bins in the root endosphere (Fig. 4a; Table S1 in Supporting Information). Among the bins recovered from the root endosphere, three bins belonged to Actinobacteria, and three bins belonged to Alphaproteobacteria. In the bins recovered from the rhizosphere, four bins were assigned to Actinobacteria, four bins to Alphaproteobacteria, and eight bins to Gammaproteobacteria. Notably, five bins in the root endosphere belonged to the same genus or species as bins in the rhizosphere, which were assigned to *Rhizorhapis*, *Sphingopyxis*, *Pseudolysinimonas*, and *Chryseobacterium*.

The biosynthetic gene clusters of the metagenome-assembled genomes were predicted by antiSMASH. The results indicated that Actinobacteria and Alphaproteobacteria harbored more diverse secondary metabolites than other taxa (Fig. 4b). Particularly, Actinobacteria was found to have a higher number of NRPS, T1PKS (Type 1 PolyKetide Synthase), and terpene, while Alphaproteobacteria was found to have a higher number of T3PKS (Type 3 PolyKetide Synthase) and siderophores (including NRPS-independent siderophore and NPR metallophore).

## DISCUSSION

In this study, we conducted a comparative analysis of the endophytic and epiphytic microbiota in tobacco (*Nicotiana tabacum*). Our results reveal that Actinobacteria and Alphaproteobacteria were more abundant in the root endosphere, accompanied by a range of functional orthologs, than in the rhizosphere. Notably, our genome-resolved metagenomics approach indicates that Actinobacteria and Alphaproteobacteria exhibited a higher diversity of secondary metabolites as characteristic features.

A previous study in the Cariaco Basin provided evidence that the microbial lifestyle, distinguishing between particle-associated and free-living microbes, is linked to variations in secondary metabolites (47). It was found that taxa associated with particles expressed a higher number of secondary metabolites than those in the free-living mode. In line with this study, our results also revealed a greater diversity of secondary metabolites within the enriched taxa, suggesting a correlation between the diversity of secondary metabolites and the endophytic lifestyle. These secondary metabolites may play important roles in plant-microbe interactions. For instance, several secondary metabolites, responsible for plant defense, are produced by the endophytic microorganism to escape host defenses, allowing them to reside within the plant without eliciting immune responses (48).

In the assembled genomes assigned to Actinobacteria, we identified two prominent classes: NRPS and PKS. This finding was consistently supported by the enrichment of antibiotic synthesis KOs within Actinobacteria, primarily represented by polyketide synthase and non-ribosomal peptide synthetase, indicating that antibiotic synthesis may confer a competitive advantage to Actinobacteria. Furthermore, we detected the presence of secondary metabolites, specifically NRPS-independent siderophores, in Actinobacteria. On the other hand, the enriched pyochelin biosynthesis KOs, associated with Actinobacteria, were classified as NRPS-dependent siderophores (49, 50). As iron is often a limiting growth factor for bacteria (51, 52), the diverse strategy for iron acquisition likely contributes to Actinobacteria’s ability to thrive in the root endosphere. In addition, our results revealed an enrichment of plant cell-wall-degrading enzymes in the root endosphere, originating from Actinobacteria. Previous studies have highlighted the wide range of enzymes possessed by Actinobacteria capable of breaking down plant cell walls (53), which is essential for their capacity to decompose plant material. This ability to break down complex plant cell wall structures likely facilitates their colonization of the plant endosphere.

Regarding the genomes assigned to Alphaproteobacteria, our analysis revealed that terpene and homoserine lactone (AHLs) were the most prevalent secondary metabolites. AHLs, produced by bacteria, play a distinctive role in modulating the expression of plant defense genes and are critical to plant-bacteria interactions (54, 55). The enrichment of Alphaproteobacteria aligns with previous research, indicating their common presence as common root-associated bacteria (9). Interestingly, we also observed an enrichment of genes encoding type IV secretion systems (T4SS) in the endosphere. T4SS are specialized protein transport systems typically associated with the transfer of effector proteins to host cells in pathogenic bacteria (56). However, T4SS are also known to be involved in beneficial plant-microbe interactions, facilitating the transfer of beneficial traits from soil bacteria to plant hosts (57). Indeed, previous studies have demonstrated the importance of T4SS in the colonization of legume root nodules by rhizobia, which are responsible for fixing atmospheric nitrogen for their plant hosts (58). In fact, T4SS have been shown to be important for the colonization of the legume root nodules by rhizobia, which fix atmospheric nitrogen for their plant hosts (58). This suggests that Alphaproteobacteria may employ T4SS to establish beneficial interactions with plant hosts in the root endosphere. Nevertheless, further research is needed to elucidate the specific roles of T4SS in Alphaproteobacteria-mediated plant-microbe interactions.

Remarkably, we observed a significant enrichment of nicotine-degrading enzymes in the root endosphere, originating from Actinobacteria and Alphaproteobacteria. Nicotine, a toxic compound, can limit the growth of many microorganisms. The ability to degrade nicotine potentially provides these bacteria with a competitive edge, enabling them to establish and thrive in the root endosphere of tobacco plants. It is also possible that the enrichment of nicotine-degrading bacteria in the root endosphere is a result of co-evolution between the plant and its associated microbiome. Nicotine is a secondary metabolite produced by tobacco plants as a defense mechanism against herbivores and pathogens (59). The presence of bacteria with nicotine-degrading genes may have co-evolved with the plant as a means of neutralizing this defense mechanism and establishing a mutualistic relationship.

In summary, the enrichment of bacteria in the root endosphere of plants with a high diversity of secondary metabolites can influence plant-microbe interactions in various ways. The enrichment could be a result of both plant selection and the competitive advantage conferred on the microbes. Further research is needed to better understand the mechanisms by which endophytes interact with plants.

## Data Availability

The amplicon and metagenomic sequencing data were deposited in the GSA database (https://bigd.big.ac.cn/gsa/) under the accession numbers CRA008961 and CRA013345, respectively.

## SUPPLEMENTAL MATERIAL

Supplemental material msphere.00607-23-s0001.docx Supplemental methods, Figure S1, Figure S2, and Table S1.

ASM does not own the copyrights to Supplemental Material that may be linked to, or accessed through, an article. The authors have granted ASM a non-exclusive, world-wide license to publish the Supplemental Material files. Please contact the corresponding author directly for reuse.
