# Supplementary material for: Taxonomic and functional assembly cues enrich the endophytic tobacco microbiota across epiphytic compartments
Source: mSphere. 2023 Dec 12;9(1):e00607-23. doi: 10.1128/msphere.00607-23 (PMC10826349; doi:10.1128/msphere.00607-23)
Supplement: Supplemental material — Supplemental methods, Figure S1, Figure S2, and Table S1. [file msphere.00607-23-s0001.docx]

**Supporting Information**

**Taxonomic and Functional Assembly Cues Enriching Endophytic Tobacco Microbiota across Epiphytic Compartments**

Luhua Yang^1,2^, Yuan Guo^3^, Hui Yang^3,5,6^, Shun Li ^1,2^, Yunzeng Zhang^7^, Likai Hao^3,4,5*^

^1^ Key Laboratory of Urban Environment and Health, Ningbo Urban Environment Observation and Research Station, Institute of Urban Environment, Chinese Academy of Sciences, Xiamen 361021, PR China

^2^ Zhejiang Key Laboratory of Urban Environmental Processes and Pollution Control, CAS Haixi Industrial Technology Innovation Center in Beilun, Ningbo 315830, PR China

^3^ State Key Laboratory of Environmental Geochemistry, Institute of Geochemistry, Chinese Academy of Sciences, Guiyang 550081 PR China

^4^ CAS Center for Excellence in Quaternary Science and Global Change, Xi’an 710061 PR China

^5^ University of Chinese Academy of Sciences, Beijing 100049 PR China

^6^ Guizhou Academy of Tobacco Science, Guiyang 550081, China

^7^ College of Bioscience and Biotechnology, Yangzhou University, Yangzhou 225009 PR China

*Corresponding author: Likai Hao, [haolikai@mail.gyig.ac.cn](mailto:haolikai@mail.gyig.ac.cn)

This file include:

Supplementary methods

Figure S1

Figure S2

Table S1

**Supplementary methods**

The PCR reactions of the amplicon library contained 25 μl 2x Premix Taq (Takara Biotechnology, Japan), 1 μl each primer (10 μM) and 3 μl DNA (20 ng/μl) template in a volume of 50 µl. The thermocycling conditions were: 5 min at 94 °C for initialization; 30 cycles of 30 s denaturation at 94 °C, 30 s annealing at 52 °C, and 30 s extension at 72 °C; followed by 10 min final elongation at 72 °C. Sequencing libraries were generated using NEBNext® Ultra™ II DNA Library Prep Kit for Illumina® (New England Biolabs, USA) following the manufacturer's instruction. The quality was assessed on the Qubit@ 2.0 Fluorometer (Thermo Fisher Scientific, USA)

Fig. S1 The phylogenetic relationship of the three tobacco varieties. The variety K326 is the parent of the variety Y87. The variety Y87 is the parent of the variety Y28. The varieties sampled in this study were in solid line, while the varieties not used in this study were in dashed line.

Fig. S2 The potential sources of tobacco associated bacterial communities in each compartment. The percentage indicated the proportion coming from the known sources.

Table S1 The metagenome-assembled bins retrieved from the (a) rhizosphere and (b) root endosphere

(a) The metagenome-assembled bins retrieved from the rhizosphere

| ID | Source | Phylum | Class | Order | Family | Genus | Species | Complete-ness | Contami-nation | size | N50 |
| --- | --- | --- | --- | --- | --- | --- | --- | --- | --- | --- | --- |
| Bin1 | K326 | Actinobacteriota | Actinomycetia | Actinomycetales | Microbacteriaceae | Pseudolysinimonas |  | 79.58 | 1.26 | 1485655 | 5759 |
| Bin2 | Y87 | Actinobacteriota | Actinomycetia | Mycobacteriales | Mycobacteriaceae | Antrihabitans | Antrihabitans stalactiti | 70.32 | 4.09 | 3966491 | 4247 |
| Bin3 | Y87 | Actinobacteriota | Actinomycetia | Mycobacteriales | Micromonosporaceae | Glycomyces |  | 93.44 | 1.75 | 4342710 | 9016 |
| Bin4 | co | Actinobacteriota | Actinomycetia | Propionibacteriales | Nocardioidaceae | Marmoricola |  | 89.34 | 1 | 2632159 | 18112 |
| Bin5 | Y28 | Bacteroidota | Bacteroidia | Chitinophagales | Chitinophagaceae | Chitinophaga | Chitinophaga sp018224885 | 95.57 | 0.99 | 7606416 | 27436 |
| Bin6 | K326 | Bacteroidota | Bacteroidia | Flavobacteriales | Weeksellaceae | Chryseobacterium | Chryseobacterium wanjuense | 84.33 | 0.41 | 2553891 | 4917 |
| Bin7 | K326 | Bacteroidota | Bacteroidia | Flavobacteriales | Weeksellaceae | Chryseobacterium |  | 80.25 | 9.81 | 2162418 | 3031 |
| Bin8 | Y87 | Nitrospirota | Nitrospiria | Nitrospirales | Nitrospiraceae |  |  | 74.41 | 5.68 | 2071125 | 6817 |
| Bin9 | Y28 | Nitrospirota | Nitrospiria | Nitrospirales | Nitrospiraceae | Nitrospira_C |  | 91.56 | 5.51 | 3454169 | 15371 |
| Bin10 | co | Methylomirabilota | Methylomirabilia | Rokubacteriales | CSP1-6 | AR37 |  | 71.78 | 5.6 | 3488651 | 5345 |
| Bin11 | co | Patescibacteria | Saccharimonadia | Saccharimonadales | UBA4665 | JACMPA01 |  | 71.29 | 0 | 1142538 | 171784 |
| Bin12 | K326  /Y87 | Patescibacteria | Saccharimonadia | UBA4664 | UBA4664 | UBA5169 |  | 77.69 | 1.19 | 1090633 | 33298 |
| Bin13 | K326  /Y87 | Patescibacteria | Saccharimonadia | UBA4664 | UBA4664 | UBA5169 |  | 75.25 | 0.5 | 971776 | 27930 |
| Bin14 | Y87 | Proteobacteria | Alphaproteobacteria | Rhizobiales | Rhizobiaceae | Agrobacterium | Agrobacterium fabrum | 90.81 | 4.24 | 5314953 | 12425 |
| Bin15 | K326  /Y87 | Proteobacteria | Alphaproteobacteria | Sphingomonadales | Sphingomonadaceae | Novosphingobium |  | 97.85 | 1.67 | 3055512 | 17894 |
| Bin16 | Y87 | Proteobacteria | Alphaproteobacteria | Sphingomonadales | Sphingomonadaceae | Sphingopyxis |  | 90.99 | 4.81 | 3712865 | 14903 |
| Bin17 | co | Proteobacteria | Alphaproteobacteria | Sphingomonadales | Sphingomonadaceae | Rhizorhapis |  | 94.63 | 1.4 | 2920672 | 28067 |
| Bin18 | K326  /Y28 | Proteobacteria | Gammaproteobacteria | Burkholderiales | Rhodocyclaceae |  |  | 72 | 3.48 | 2145991 | 10059 |
| Bin19 | Y87 | Proteobacteria | Gammaproteobacteria | Burkholderiales | Burkholderiaceae | Albitalea |  | 74.94 | 3.83 | 2800556 | 5148 |
| Bin20 | K326 | Proteobacteria | Gammaproteobacteria | Burkholderiales | Burkholderiaceae | Achromobacter | Achromobacter mucicolens | 92.58 | 5.46 | 5379377 | 11830 |
| Bin21 | K326 | Proteobacteria | Gammaproteobacteria | Enterobacterales | Enterobacteriaceae | Enterobacter | Enterobacter asburiae_B | 70.33 | 8.76 | 2705335 | 2510 |
| Bin22 | Y87 | Proteobacteria | Gammaproteobacteria | Pseudomonadales | Pseudomonadaceae | Pseudomonas_E | Pseudomonas_E sp902506495 | 99.84 | 2.85 | 5183439 | 100710 |
| Bin23 | K326  /Y87 | Proteobacteria | Gammaproteobacteria | Pseudomonadales | Pseudomonadaceae | Pseudomonas_E | Pseudomonas_E bijieensis | 72.9 | 7.71 | 3825215 | 7705 |
| Bin24 | co | Proteobacteria | Gammaproteobacteria | Pseudomonadales | Spongiibacteraceae |  |  | 92.78 | 3.42 | 4160291 | 10381 |
| Bin25 | Y28 | Proteobacteria | Gammaproteobacteria | Xanthomonadales | Rhodanobacteraceae | Rhodanobacter | Rhodanobacter sp001898415 | 70.26 | 8.92 | 2338260 | 4097 |

(b) The metagenome-assembled bins retrieved from the root endosphere

| ID | Source | Phylum | Class | Order | Family | Genus | Species | completeness | contamination | size | N50 |
| --- | --- | --- | --- | --- | --- | --- | --- | --- | --- | --- | --- |
| Bin1 | k326/Y87 | Actinobacteriota | Actinomycetia | Actinomycetales | Microbacteriaceae | Pseudolysinimonas |  | 83.61 | 0.73 | 1559484 | 7116 |
| Bin2 | Y87 | Actinobacteriota | Actinomycetia | Mycobacteriales | Pseudonocardiaceae | Amycolatopsis |  | 100 | 1.41 | 8722375 | 23660 |
| Bin3 | Y87/Y28 | Actinobacteriota | Actinomycetia | Mycobacteriales | Pseudonocardiaceae | Pseudonocardia |  | 72.26 | 4.02 | 5639006 | 5387 |
| Bin4 | Y87 | Bacteroidota | Bacteroidia | Flavobacteriales | Weeksellaceae | Chryseobacterium |  | 71.95 | 3.48 | 1973011 | 2891 |
| Bin5 | Y87 | Bacteroidota | Bacteroidia | Flavobacteriales | Weeksellaceae | Chryseobacterium | Chryseobacterium wanjuense | 94.1 | 0.26 | 3229495 | 8275 |
| Bin6 | Y87 | Proteobacteria | Alphaproteobacteria | Rhizobiales | Rhizobiaceae | Ensifer | Ensifer adhaerens | 70.18 | 9.69 | 4706990 | 3929 |
| Bin7 | K326/Y87 | Proteobacteria | Alphaproteobacteria | Sphingomonadales | Sphingomonadaceae | Rhizorhapis |  | 96.18 | 0.74 | 2508081 | 22510 |
| Bin8 | K326/Y87 | Proteobacteria | Alphaproteobacteria | Sphingomonadales | Sphingomonadaceae | Sphingopyxis |  | 91.26 | 3.8 | 3736334 | 15844 |
